# Forward Looking Radar Imaging by Truncated Singular Value Decomposition and Its Application for Adverse Weather Aircraft Landing

**DOI:** 10.3390/s150614397

**Published:** 2015-06-18

**Authors:** Yulin Huang, Yuebo Zha, Yue Wang, Jianyu Yang

**Affiliations:** School of Electronic Engineering, University of Electronic Science and Technology of China, 2006 Xiyuan Road, Gaoxin Western District, Chengdu 611731, China; E-Mails: yulinhuang@uestc.edu.cn (Y.H.); wyue_uestc@126.com (Y.W.); jyyang@uestc.edu.cn (J.Y.)

**Keywords:** aircraft landing, radar imaging, truncated singular value decomposition, deconvolution

## Abstract

The forward looking radar imaging task is a practical and challenging problem for adverse weather aircraft landing industry. Deconvolution method can realize the forward looking imaging but it often leads to the noise amplification in the radar image. In this paper, a forward looking radar imaging based on deconvolution method is presented for adverse weather aircraft landing. We first present the theoretical background of forward looking radar imaging task and its application for aircraft landing. Then, we convert the forward looking radar imaging task into a corresponding deconvolution problem, which is solved in the framework of algebraic theory using truncated singular decomposition method. The key issue regarding the selecting of the truncated parameter is addressed using generalized cross validation approach. Simulation and experimental results demonstrate that the proposed method is effective in achieving angular resolution enhancement with suppressing the noise amplification in forward looking radar imaging.

## 1. Introduction

The demand of civil aviation transportation in the all-time and all-weather has increased significantly since there is often the case that aircraft departure from one hub are delayed due to adverse weather, which results in many possible direct and indirect cost impacts. If there is known to be poor visibility at the destination region, the aircraft take off may be cancelled or delayed, disrupting the original flight schedule as well as impacting the scheduling of other planned flights [1]. Poor visibility is generally the result of fog, but other inclement weather conditions, such as rain, snow, sleet, dust storms or smoke, can also restrict visibility in the surrounding environment [2]. Several systems are designed to enhance situational awareness by providing supplemental visual data to the pilot, which includes runways, landing approach markers, other aircrafts and other terrain. These features could not be seen during night and low visibility conditions. There is a clear incentive consideration in providing sufficient visibility range or in generating a normal looking image in adverse weather using optical or radar sensor systems. Many of the achievements of aircraft landing problem have been accomplished to aid aircraft's takeoff and landing in adverse weather. A synthetic vision method for enhancing poor visibility flight operations can be found in [3], but the optical sensor generated imagery can not provide the sufficient visibility range in adverse weather. In [4], the authors developed a procedure for topographic terrain mapping, which is used for aircraft landing. Despite the progress in radar imaging methods, this method do not sufficiently detect slight angular changes of the targets in the image scene.

One of the most promising solutions to the problem is to use a forward looking scanning radar system that provides supplemental visual data to allow pilots to see through fog and other adverse weather, which has been widely used in many military and civilian fields, including remote sensing [5], flight navigation [6] and cargo airdropping [7], etc. The rationale behind the forward looking radar imaging is that utilizes the return power seen by the antenna beam to make image of the ground as the antenna beam scans the areas. To realize these applications, high resolution of the two dimensions for forward looking radar imagery is essential. The high range resolution can be achievable by transmitting the wideband signal and using the pulse compression technique. However, the angular resolution of forward looking scanning radar image is relatively poor when compared with the achievable range resolution. For forward looking scanning radar system, the angular resolution enhancement capabilities are physically limited by the effective wavelength and the physic size of antenna aperture. Therefore, the improvement in angular resolution of scanning radar image can be accomplished by either increasing the physical size of radar antenna, or by increasing the frequency of the transmitted signal. However, neither of these solutions were attractive. This demands use of signal processing techniques to process the acquired echo data and obtain improved angular resolution in scanning radar image. This kinds of techniques are called angular super-resolution algorithms in this paper. The main motivation behind the angular super-resolution lies in using mathematical processing methods to increase the angular resolution beyond the limitation of radar system parameters.

According to [8], the received signal *g* can be modeled by *g* = *H* * *f* + *n*, and our objective is to recover *f* from *g*, where * stands for the convolution operator, *f* is reflectivity of the scatter, *n* denotes noise, and *H* represents a convolution matrix acting on the *f*. Therefore, deconvolution method can enhance the angular resolution of a forward looking scanning radar imagery in theory [9,10]. Deconvolutiuon problem is physically considered as the analog of a linear inversion task, which is formulated and solved in [11]. Recently, applications of denconvolution can be found in [12–14] for imaging processing, [15–17] for microwave imaging, [18,19] for MRI and CT. Deconvolution method also has applications in radar imaging; see [20,21]. These deconvolution methods can be divided into two categories: classic and Bayesian approaches. Both classic (see [22–26]) and Bayesian (see [27–29]) approaches for solving the problem are posed as a minimization of a cost function having the general form

(1) $$\underset{f}{\arg\min}\left\{\Phi_{\text{fit}}\left(g,f\right)+\lambda \Phi_{\text{reg}}\left(f\right)\right\}$$

where Φ*_fit_* (*g*, *f*) is minimized over a selected norm and a penalization term Φ_*reg*_(*f*) is added in an amount controlled by a tuning parameter λ to encourage or discourage certain solution based on a prior assumptions about the desirable solution characteristics. The Φ*_fit_* (*g, f*) stands for the phenomenological cost, while the Φ_*reg*_(*f*) denotes the presumptive cost [30]. There are two challenges in these deconvolution methods. One is how to choose the prior information of the object being imaged. Another challenge is that the precision of the result of the deconvolution problem depends on regularization parameter, which controls the performance of angular super-resolution versus the noise amplification.

To address these challenges, the singular value decomposition (SVD) method is introduced. The conceptual intuition of the SVD facilities its increasing use in the theoretical analysis of deconvolution problem. For microwave imaging reconstruction, the SVD method is extensive used [31-34]. Truncated singular value decomposition (TSVD) is a popular method for solving the deconvolution problem. Recently applications of TSVD method can be found in [35] for inverse scattering problem, [11] for improving the spatial resolution of radiometer data, and [36] for image restoration. But little work on TSVD method for improving the angular resolution in forward looking radar imaging has been reported. This paper presents an angular superresolution algorithm that uses truncated singular value decomposition to solve the deconvolution problem corresponding to the scanning radar imaging task.

In this paper, we present an angular super-resolution method for adverse weather aircraft landing using the truncated singular value decomposition method, which is originally developed for solving the deconvolution problem with suppressing the noise amplification in the solution [37]. To this end, we first convert the angular super-resolution problem in forward looking radar imaging task into the corresponding deconvolution problem. It is well known that the deconvolution problem is ill-posed, which means the solution to the deconvolution problem is unstable and sensitive to the noise. To address this challenge, the truncated singular value decomposition method is used to solve the deconvolution problem, which is able to realize the angular super-resolution imaging in forward looking imaging for adverse weather aircraft landing. The rational behind the noise amplification is that the large errors in the solution come from the noise singular value decomposition components associated with the small singular of the convolution matrix. This leads to a compromise between these two factors must be considered. Fortunately, the TSVD method is able to chop off those SVD components that are dominated by noise. A key issue of TSVD method is the choice of the truncation parameter. This issue is beyond the scope of this paper and the method of selecting this parameter is provided by the generalized cross-validation criterion [37,38]. Simulations and experimental results are given to demonstrate the validity of the presented method for angular super-resolution imaging in forward looking scanning imaging, which can be used for adverse weather aircraft landing.

The remainder of this paper is organized as follows: In Section 2, we provide a theoretical background of angular super-resolution imaging in forward looking scanning radar imaging for aircraft landing. In Section 3, the truncated singular value decomposition for angular super-resolution imaging in forward looking scanning radar and the generalized cross-validation method for choosing truncated parameter are presented. Section 4 is dedicated to simulation and experimental results. Finally, in Section 5, we draw our conclusion.

## 2. Theoretical Background

In this paper, we focus our attention to angular resolution enhancement in forward looking scanning radar image for adverse weather aircraft landing by solving the corresponding deconvolution problem using truncated singular value decomposition method. To this end, we first formulate the signal model of forward looking scanning radar imaging, which is also a foundation of the application of the angular super-resolution method for adverse weather aircraft landing.

**Figure 1:**
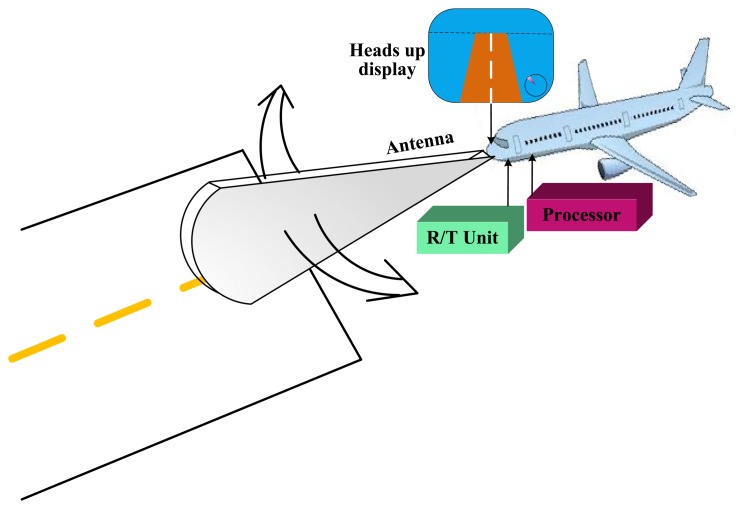
The diagram of scanning radar for aircraft landing.

As shown in Figure 1, the forward looking scanning radar system consists of an antenna, radar receiver and transmitter (R/T) display processor and heads up display. The radar platform is moving along a runway corresponding to range direction, while the antenna scans along the vertical direction of runway corresponding to azimuth/angular direction with a constant angular velocity *ω*.

**Figure 2:**
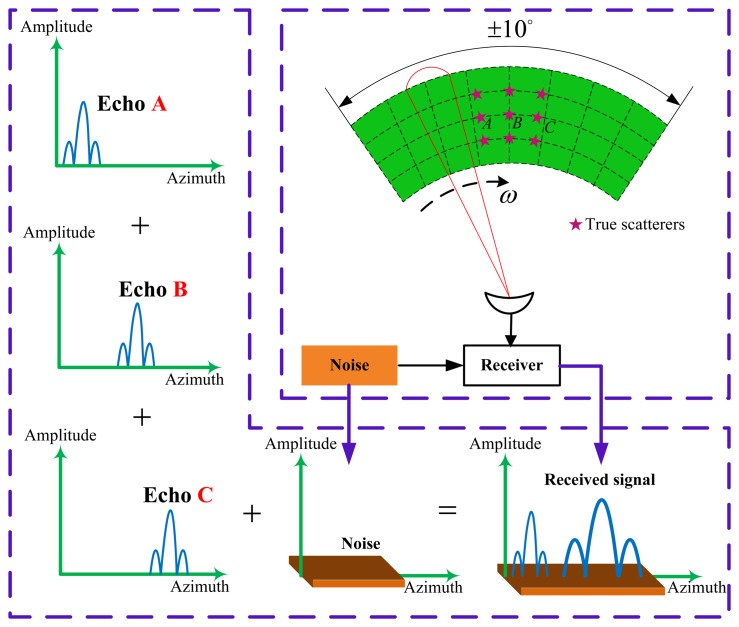
The diagram of signal model of forward looking radar.

The diagram of the signal model of the forward looking scanning radar is illustrated in the upper-right of Figure 2. This is a top down view of the antenna scan pattern and the targets located at the same range cell with different azimuth cells. Assuming that the target in the observed scene can be considered as an ideal point target. The antenna scans the targets with a constant angular velocity *ω* and then receives the echo data from the observed scene. As the antenna beam scans a point target located at a particular angular, the received output is the impulse response of the corresponding to antenna pattern. We can see from the bottom of Figure 2 that when the target B and C are close enough, the echo of these two targets are proportional to two replicas of the antenna pattern, overlapped and added to get a composite response. The resulting low resolution signal is shown in the lower right corner of Figure 2.

According to [8], the limitation of angular resolution is determined by beam width of the antenna pattern. This phenomenon brings a great difficulty in realizing the angular super-resolution imaging in forward looking scanning radar. Our primal aim in this work is to process the acquired echo data and obtain improved angular resolution via signal processing technique that breaks through the physical limitation of radar system. To reach this goal, we first introduce the signal model of the scanning radar, which is the foundation of the following discussion.

At first, the conventional range compression and range migration correction are applied to the echo data with current approaches [20,39]. The echo data after range compression and range cell migration is denoted by *g*(*θ, φ, r*) where *r* denotes the slant range from the radar to the target and *θ* represents the angle between the direction of the antenna to the target and the flight direction, and *φ* stands for the incident angle of the antenna beam. On the other hand, it is assumed that the antenna pattern is of relative isotropy to the imaging scene. Then, the received signal of forward looking radar along the range profile can be assumed as a convolution kernel comprising the antenna power pattern in the angular coordinates and the pulse modulation function in the range direction, which could be expressed as

(2) $$g\left(r,\theta ,\varphi \right)=f\left(r,\theta ,\varphi \right)*\left[a\left(\varphi ,\theta \right)x\left(\frac{2r}{c}\right)\right]$$

where * represents the convolution operator, *g*(*r*, *θ*, *φ*) is the received echo data after range compression and range cell migration, *f*(*r*, *θ*, *φ*) is the effective scattering coefficient, *a*(*φ*, *θ*) is the antenna pattern, $x\left(\frac{2r}{c}\right)$ stands for the pulse modulation function and *c* denotes the speed of light.

For mathematical simplicity, we only consider the azimuth variation. In addition, the presence of the noise makes the data *g*(*θ*) contaminated. According to [40], the echo data *g* (*θ*) can be modeled as a convolution of the antenna beam *h* (*θ*) with the reflectivity of the observed scene *f* (*θ*) plus the noise *n*(*θ*). Then, we can rewrite Equation (2) as

(3) g(θ)=f(θ)∗h(θ)+n(θ)

where *n*(*θ*) represents the noise.

From Equation (3), we have

(4) g=Hf+n

where

(5) $$g=\left[\begin{array}{c} g\left(\theta_{1}\right)\\ \vdots \\ g\left(\theta_{k}\right) \end{array}\right]\;;n=\left[\begin{array}{c} n\left(\theta_{1}\right)\\ \vdots \\ n\left(\theta_{k}\right) \end{array}\right]$$

(6) $$f={\left[f\left(\theta_{-m+1}\right),f\left(\theta_{-m+2}\right),\cdots ,f\left(\theta_{0}\right),f\left(\theta_{1}\right),\cdots ,f\left(\theta_{k}\right),f\left(\theta_{k+1}\right),\cdots f\left(\theta_{k-m+1}\right),f\left(\theta_{k+m}\right)\right]}^{T}$$

and

(7) $$H=\left[\begin{array}{cccccccccc} h\left(\theta_{m}\right) & \cdots & h\left(\theta_{0}\right) & \cdots & h\left(\theta_{-m}\right) & & & & & \\ & h\left(\theta_{m}\right) & \cdots & h\left(\theta_{0}\right) & \cdots & h\left(\theta_{-m}\right) & & & 0 & \\ & & \ddots & \ddots & \ddots & \ddots & \ddots & & & \\ & & & \ddots & \ddots & \ddots & \ddots & \ddots & & \\ 0 & & & & h\left(\theta_{m}\right) & \ddots & \ddots & \ddots & h\left(\theta_{-m}\right) & \\ & & & & & h\left(\theta_{m}\right) & \ddots & \ddots & \ddots & h\left(\theta_{-m}\right) \end{array}\right]$$

**Figure 3:**
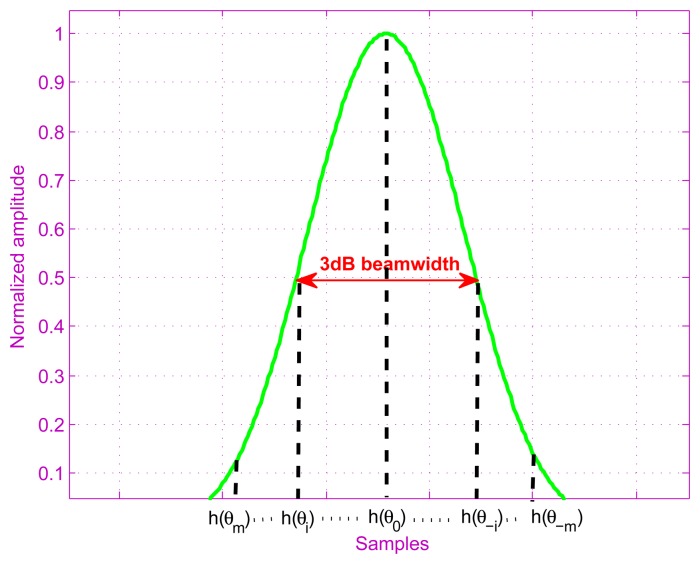
Antenna pattern.

The superscript *T* in Equation (6) is used for the matrix-vector transposition. The sequence *h*(*θ_m_*) ⋯ *h*(*θ*_0_) ⋯ *h*(*θ*_−_*_m_*) is a sampling of the antenna pattern, which is shown in Figure 3.

The forward looking scanning radar imaging for aircraft landing task is to recover [*f* (*θ*_1_), ⋯ , *f* (*θ_k_*)]*^T^* given the convolution matrix *H* and the echo data *g* = [*g*(*θ*_1_), ⋯ ,*g*(*θ_k_*)] of finite length. However, the echo data *g* is determined not only by [*f*(*θ*_1_), ⋯ , *f*(*θ_k_*)]*^T^*, but also by Equation (6) and the system Equation (4) is underdetermined. To overcome this problem, some prior information about the observed scene should be incorporated. According to [41], we should make the assumptions on the values of the [*f*(*θ*_1_), ⋯ , *f*(*θ_k_*)]*^T^* outside the domain where *g* is determined. In this paper, we assume that the true scene outside the field of view is a mirror reflection of the scene within the field of observation. This leads to that the convolution matrix is a Toeplitz-plus-Hankel matrix. More precisely, the convolution matrix *H* in Equation (4) can be written as follows:

(8) $$H=\left[\begin{array}{ccccc} h\left(\theta_{0}\right) & \cdots & h\left(\theta_{-m}\right) & \cdots & 0\\ \vdots & \ddots & \ddots & \ddots & \\ h\left(\theta_{m}\right) & \ddots & \ddots & \ddots & h\left(\theta_{-m}\right)\\ & \ddots & \ddots & \ddots & \vdots \\ 0 & & h\left(\theta_{m}\right) & \ddots & h\left(\theta_{0}\right) \end{array}\right]+\left[\begin{array}{ccc} h\left(\theta_{1}\right) & \cdots & h\left(\theta_{m}\right)\\ \vdots & ⋰ & \\ h\left(\theta_{m}\right) & & \end{array}\right]+\left[\begin{array}{ccc} & & h\left(\theta_{-m}\right)\\ & ⋰ & \vdots \\ h\left(\theta_{-m}\right) & \cdots & h\left(\theta_{-1}\right) \end{array}\right]$$

For an area target scene, the reflectivity coefficients of the scatterers can be denoted as a 2-D matrix

(9) $$F=\left[\begin{array}{c} F_{-Q}\\ \vdots \\ F_{0}\\ \vdots \\ F_{Q} \end{array}\right]={\left[\begin{array}{ccccc} f\left(r_{-Q},\theta_{-A}\right) & \cdots & f\left(r_{-Q},\theta_{0}\right) & \cdots & f\left(r_{-Q},\theta_{A}\right)\\ \vdots & \ddots & \vdots & \ddots & \vdots \\ f\left(r_{0},\theta_{-A}\right) & \cdots & f\left(r_{0},\theta_{0}\right) & \cdots & f\left(r_{0},\theta_{A}\right)\\ \vdots & \ddots & \vdots & \ddots & \vdots \\ f\left(r_{Q},\theta_{-A}\right) & \cdots & f\left(r_{Q},\theta_{0}\right) & \cdots & f\left(r_{Q},\theta_{A}\right) \end{array}\right]}_{\left(2Q+1\right)\times \left(2A+1\right)}$$

where *f*(*r_q_*, *θ_a_*)(*q* = −*Q*, ⋯, 0, ⋯, *Q*; *a* = −*A*, ⋯, 0, ⋯ , *A*) represents the discrete equivalent reflectivity coefficient at the *q*-th position of the runway and the *a*-th position along the vertical direction of the runway, 2*Q*+1 is the number of points along the runway, and 2*A*+1 is the number of points along the vertical direction of the runway after the discretization of the scene. The results of aforementioned can be extended to area target angular super-resolution imaging. In this case, the convolution matrix *H* corresponding in Equation (4) is a block Toeplitz-plus-Hankel matrix with Toeplitz-plus Hankel blocks, which can be expressed as

(10) $$H=\left[\begin{array}{ccccc} H^{\left(0\right)} & \cdots & H^{\left(-Q\right)} & \cdots & 0\\ \vdots & \ddots & \ddots & \ddots & \\ H^{\left(Q\right)} & \ddots & \ddots & \ddots & H^{\left(-Q\right)}\\ & \ddots & \ddots & \ddots & \vdots \\ 0 & & H^{\left(Q\right)} & \ddots & H^{\left(0\right)} \end{array}\right]+\left[\begin{array}{ccccc} H^{\left(1\right)} & \cdots & H^{\left(-Q\right)} & & 0\\ \vdots & ⋰ & & & \\ H^{\left(Q\right)} & & & & H^{\left(-Q\right)}\\ & & & ⋰ & \vdots \\ 0 & & H^{\left(-Q\right)} & \cdots & H^{\left(1\right)} \end{array}\right]$$

with each block *H*^(*i*)^ (*i* = −*Q*, ⋯, 0, ⋯, *Q*) being an (2*A*+1) × (2*A*+1) matrix of the form given in Equation (8). We note that the **f** corresponding *f* in Equation (4) can be expressed as

(11) $$\begin{array}{ll} f & =\upsilon ec\left(F^{T}\right)=\left[F_{-Q}^{T},\cdots ,F_{0}^{T},\cdots F_{Q}^{T}\right]\\ & ={\left[f\left(r_{-Q},\theta_{-A}\right),\cdots ,f\left(r_{-Q},\theta_{A}\right),\cdots ,f\left(r_{Q},\theta_{-A}\right),\cdots ,f\left(r_{Q},\theta_{A}\right)\right]}^{T} \end{array}$$

Based on Equations (5), (6), and (7), the discrete expression of the echo data of the observed scene *F* can be expressed as follows:

$$\text{g}=\left[\begin{array}{c} g\left(r_{-Q},\theta_{-A}\right)\\ \vdots \\ g\left(r_{-Q},\theta_{A}\right)\\ \vdots \\ g\left(r_{-Q},\theta_{-A}\right)\\ \vdots \\ g\left(r_{-Q},\theta_{-A}\right) \end{array}\right]=\text{Hf}+\text{n}=\left(\left[\begin{array}{ccccc} H^{\left(0\right)} & \cdots & H^{\left(-Q\right)} & \cdots & 0\\ \vdots & \ddots & \ddots & \ddots & \\ H^{\left(Q\right)} & \ddots & \ddots & \ddots & H^{\left(-Q\right)}\\ & \ddots & \ddots & \ddots & \vdots \\ 0 & & H^{\left(Q\right)} & \ddots & H^{\left(0\right)} \end{array}\right]+\left[\begin{array}{ccccc} H^{\left(1\right)} & \cdots & H^{\left(Q\right)} & & 0\\ \vdots & ⋰ & & & \\ H^{\left(Q\right)} & & & & H^{\left(-Q\right)}\\ & & & ⋰ & \vdots \\ 0 & & H^{\left(-Q\right)} & \cdots & H^{\left(1\right)} \end{array}\right]\right)\;\left[\begin{array}{c} f\left(r_{-Q},\theta_{-A}\right)\\ \vdots \\ f\left(r_{-Q},\theta_{A}\right)\\ \vdots \\ f\left(r_{Q},\theta_{-A}\right)\\ \vdots \\ f\left(r_{Q},\theta_{-A}\right) \end{array}\right]+\left[\begin{array}{c} n\left(r_{-Q},\theta_{-A}\right)\\ \vdots \\ n\left(r_{-Q},\theta_{A}\right)\\ \vdots \\ n\left(r_{Q},\theta_{-A}\right)\\ \vdots \\ n\left(r_{Q},\theta_{-A}\right) \end{array}\right]$$

Hence, the projection to obtain echo data from the observed scene is completed.

The angular super-resolution problem in forward looking radar can be converted that gives **H**, recovering **f** from **g** by solving the Equation (2). It notes that the angular super-resolution problem in forward looking radar can be physically considered as the analog of the antenna pattern **H** deconvolution. This problem is often called inverse problem, which is characterized by an ill-posed under-determined convolution matrix **H**. However, for the purpose of solving the deconvolution problem Equation (2) and consequently better angular resolution than that of the real antenna, the linear deconvolution can be stated as the task of finding a linear operator *K* = **H**^−1^ such that

(12) $$\hat{F}\left(w\right)=G\left(w\right)K\left(w\right)=F\left(w\right)+\frac{N\left(w\right)}{H\left(w\right)}$$

where **F**(*w*), **G**(*w*), **H**(*w*), and **N**(*w*) are the Fourier transforms of **f**, **g**, **H**, and **n** in Equation (2), respectively. However, this method tends to be very noise-sensitive and unstable. The reason is that convolution in the Fourier domain corresponds to multiplication while deconvolution is Fourier division. The high-frequency spectral components of the **H**(*w*) are lost due to the finite size of the antenna, which results in that the multipliers are often small for high frequencies, and the inverse filter $\frac{1}{H\left(w\right)}$ is large when **H**(*w*) is small, leading to the degradation of the angular super-resolution performance for forward looking radar image.

## 3. Truncated Singular Value Decomposition for Angular Super-Resolution

In this section, we present the truncated singular value decomposition method to realize the angular super-resolution in forward looking scanning radar imaging. To this end, we first give the details of the singular value decomposition (SVD) as a foundation of the proposed method. Then, a truncated singular value decomposition method is presented for solving the problem Equation (2) and consequently create better angular resolution in radar image than that of real beam radar image.

The use of SVD goes back to Varah [42] and the early history of SVD is described in [43]. For mathematical simplicity, considering the matrix *H* in Equation (4), the SVD is given by

(13) $$\begin{array}{c} H=U\sum V^{T}=\sum_{i=1}^{Q}u_{i}\sigma_{i}u_{i}^{T}\\ UU^{T}=VV^{T}=I_{Q}\\ \sum =\left[\begin{array}{cc} D & 0\\ 0 & 0 \end{array}\right] \end{array}$$

where *D* = *diag*(*σ*_1_, *σ*_2_, ⋯ , *σ_n_*) represents a diagonal matrix with the singular value organized in decreasing order; *U* = (*u*_1_, *u*_2_, ⋯ , *u_n_*) and *V* = (*v*_1_, *v*_2_, ⋯, *v_n_*) are respectively, the left and right singular vectors and both matrices have orthonormal columns [32].

The solution of Equation (4) can be expressed as follows:

(14) $$f_{k}=V\sum^{+}U^{T}g=f+\sum_{i=1}^{k}\frac{u_{i}^{T}n}{\sigma_{i}}\upsilon_{i}$$

where *k* is the truncation parameter and Σ^+^ is the pseudoinverse of Σ. We can see that if *σ_i_* is very small, even a small size of *σ_i_* can causes large error. It is noted that the smaller *σ_i_* values are less reliable and must be discarded [37]. However, the noise or errors in the data *g* are amplified by the inverse of the corresponding small singular values and mapped as spurious components of the object estimated [30]. This makes that an optimal truncation parameter can be found by maximizing the fidelity in Equation (1) over the truncation parameter. The rationale behind the choice of truncation parameter is to compute an approximate solution by chopping off those SVD components that are dominated by the noise.

In this paper, the generalized cross-validation (GCV) method is adopted. An advantage of GCV is that does not require prior knowledge of the variance of the noise in the model Equation (4). The GCV is described in [44,45], which has proved to be an excellent method of choosing a regularization parameter. The GCV method aims at minimizing the predictive mean-square errors as follows:

(15) $${\left\|Hf_{k}-\hat{g}\right\|}_{2}^{2}$$

where *ĝ* is noise-free echo data. In practice, the *ĝ* is unknown, it can be demonstrated [32,38] that under the hypothesis of the white noise, the parameter *k* can be obtained via minimizing the GCV fuinction:

(16) $$\text{GCV}\left(k\right)=\frac{{\left\|Hf_{k}-g\right\|}_{2}^{2}}{\text{trace}\left(I-HH_{k}^{†}\right)}$$

where $H_{k}^{†}$ satifies $H_{k}^{†}g=f_{k}$ and *trace*(·) denotes the sum of the diagonal components inside the brackets.

## 4. Simulations and Experimental Results

In this section, we focus on the angular super-resolution performance of the TSVD method. Our purpose is to demonstrate the accuracy of the TSVD method. The accuracy of TSVD method is shown mainly by comparison with the angular super-resolution method in [20]. In the following, we denote the algorithm in [20] as Guan's method.

### 4.1. Simulations

**Figure 4:**
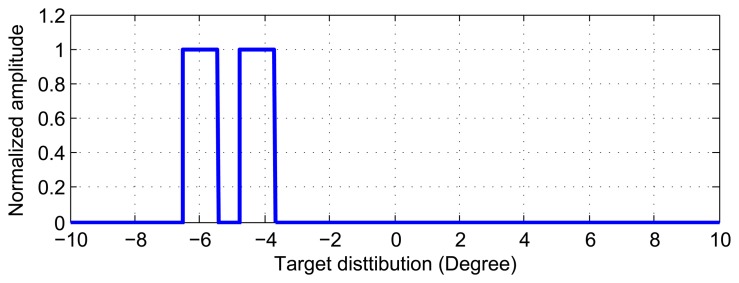
Location of targets in simulated scene.

In this subsection, we present the simulation results under different noise levels. The simulation scene is shown in Figure 4. The two targets locate at −6° and −4.2°, with their corresponding amplitude of the same value as 1. Some simulation parameters are shown in Table 1. Samples in the 3 dB beam width is about 573.

**Figure 5:**
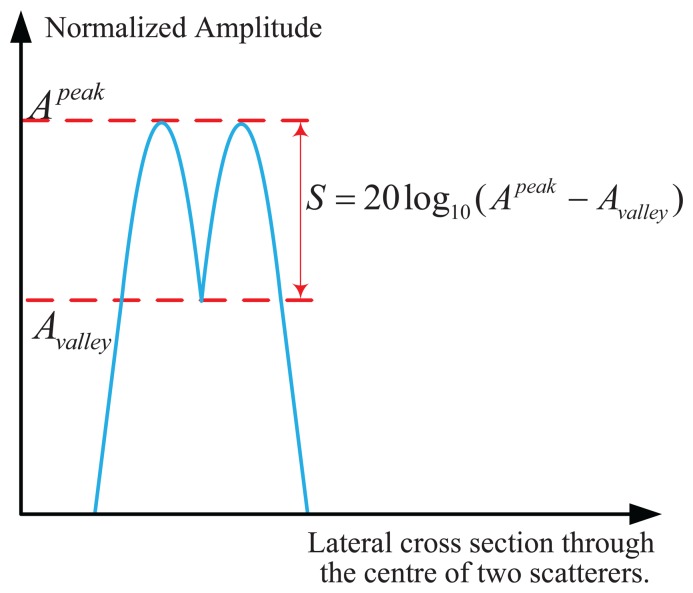
The definition of peak to valley point difference in dB.

In order to quantify and compare the angular super-resolution performance of the TSVD method on the simulation data, relative error (ReErr), structure similarity (SSIM) [46] and the peak to valley point difference in dB are used in this section. They are defined as follows:

(17) $$\text{ReErr}=\frac{{\left\|\hat{f}-f\right\|}_{2}}{{\left\|f\right\|}_{2}};\text{SSIM}=\frac{2\rho_{\left(\hat{f},f\right)}\cdot \left(2\mu_{\hat{f}}\cdot \mu_{f}\right)}{\left(\mu_{\hat{f}}^{2}+\mu_{f}^{2}\right)\left(\sigma_{\hat{f}}^{2}+\sigma_{f}^{2}\right)}$$

where *μ*, *σ*, and *ρ* are the mean, standard deviation of the vectors, and the correlations correspond to the vector *f*, *f̂*, *f̂* and *f̂* represent the obtained angular super-resolution result and the original targets, respectively. The SSIM is a quality measurement between the super-resolution result and the original The peak to valley point difference in dB is defined in Figure 5 and quantifies the ability of an angular super-resolution algorithm to separate two closely spaced targets. The difference of peak to valley point in dB is between 0 and −∞, where 0 means the angular super-resolution algorithm can fully separate two closely spaced targets.

**Figure 6:**
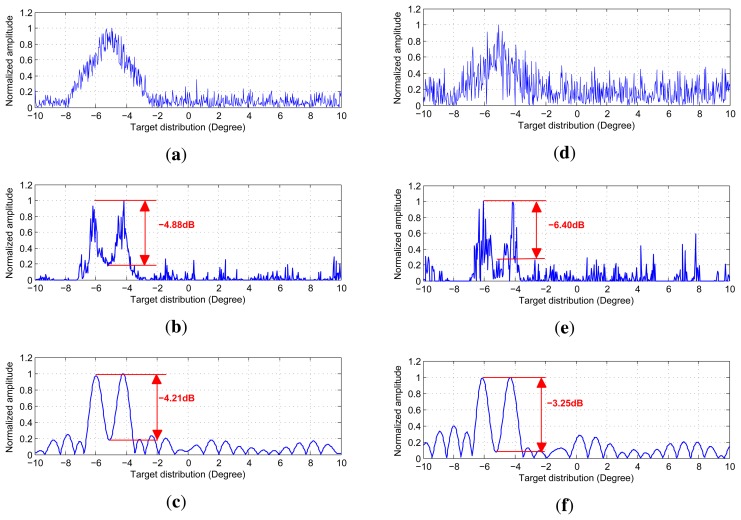
Angular super-resolution results obtained using different methods under SNR = 10 dB and 5 dB, respectively. (**a**) Echo data with noise level is 10 dB; (**b**) Angular super-resolution result of Guan's method after 15 iterations; (**c**) Angular super-resolution result of the proposed method; (**d**) Echo data with noise level is 0 dB; (**e**) Angular super-resolution result of Guan's method after 35 iterations; (**f**) Angular super-resolution results of the proposed method.

**Figure 7:**
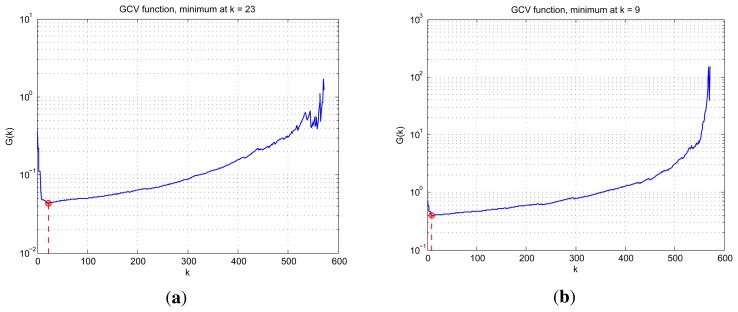
The valuation of generalized cross-validation (GCV) function versus truncation parameters with different noise levels. (**a**) SNR = 10 dB; (**b**) SNR = 0 dB.

We use the code provided by the authors of [20] to implement the angular super-resolution. The code for TSVD was coded by us. In Figure 6, we display the restored angular super-resolution radar image from different methods. The echo data with different noise levels are shown in the top row of Figure 6. From the top row Figure 6, we can see that the amplitude of the received signal is proportional to the antenna pattern. The two targets are close enough, the response of the two targets are proportional to two replicas of the antenna pattern. This phenomenon brings a great difficulty in improving the angular resolution of scanning radar. The angular super-resolution results of Guan's method and the proposed method are shown in middle and bottom row of Figure 6, respectively. The choice of truncation parameter adaptively is out scope of this paper. For the truncated parameter *k* required in proposed method, we selected it using GCV method. The improvement of the proposed method compared to Guan's method. Figure 7 shows the functional value of function with respect to the truncated parameter *k*. Figure 7a corresponds to the GCV function curve under noise level SNR = 10 dB while Figure 7b shows the GCV curve under noise level SNR = 0 dB where the truncated parameters are also presented, which is utilized in the simulation. In Figure 6, the proposed method gives the super-resolution results where the spikes of targets look fairly separated whereas in the angular super-resolution results using the Guan's method, the spikes of the targets looks more connected. The values of peak to valley difference in dB are given in the Figure 6. The difference of peak to valley in dB is between 0 and −∞, where 0 means the angular super-resolution method can fully separate two closely spaced targets. This indicates that the larger value of peak to valley in dB, the better performance of the proposed angular super-resolution method in terms of super-resolution performance.

**Figure 8:**
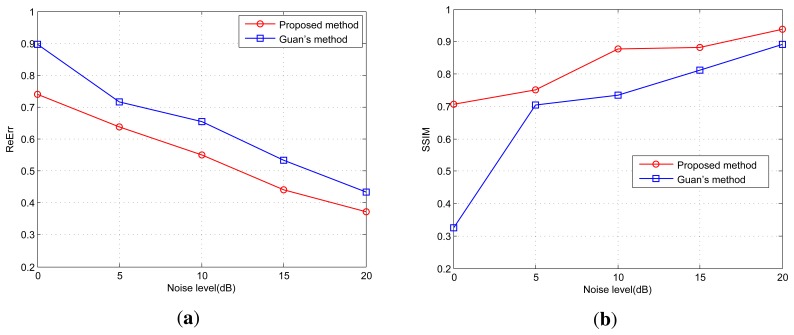
(**a**) Evolution of the relative error (ReErr) along the noise SNRs for the simulations; (**b**) Evolution of the structure similarity (SSIM) along the noise SNRs for the simulations.

In addition, the angular super-resolution results by the proposed method have both lower ReErr and and higher SSIM than that by the Guan's method. Figure 8 presents the evolution of the ReErr and the SSIM with different SNRs. Figure 8a presents the ReErr result, whose ideal value is 0. When the value of the ReErr gets smaller, the precision of the angular super-resolution method is better. The metric SSIM is a quality measurement between the angular super-resolution result and the original scene. The larger value of the SSIM, the better performance of the proposed angular super-resolution method in terms of quality. It is clear that the proposed method gives better results than Guan's method.

### 4.2. Experimental Results

**Figure 9:**
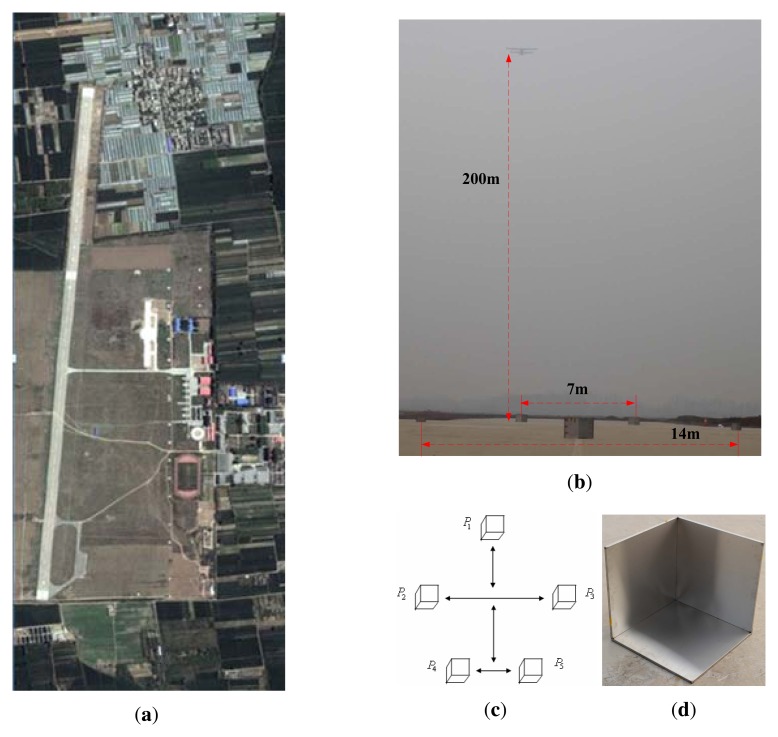
(**a**) Optical image of imaging scene; (**b**) Radar platform and five corner reflectors in the scene; (**c**) The schematic plot of the distribution of trihedral reflector; (**d**) Enlargement of trihedral reflector.

In this subsection, we present the experiment results illustrating the performance of the truncated singular value method for forward looking radar imaging and its application for adverse weather aircraft landing. Figure 9a shows the optical image of imaging scene. Figure 9b shows the position of the five corner reflectors and the distance between the reflectors. The height of the radar platform is about 200 m. The geometric relationship between the reflectors is shown in Figure 9c. Figure 9d shows the enlargement of trihedral reflector.

**Figure 10:**
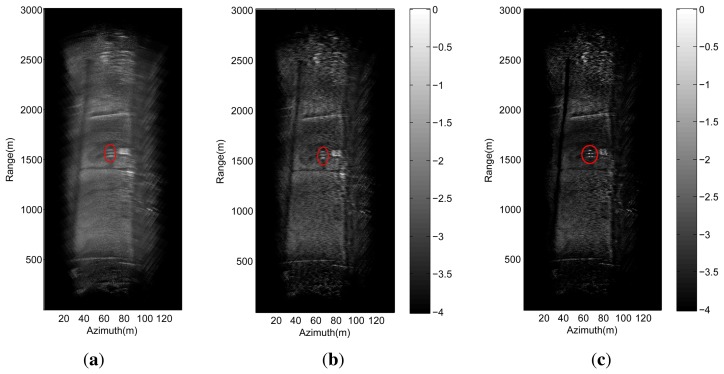
Angular super-resolution imaging results. (**a**) Real beam scanning radar imaging; (**b**) Experimental data processed by Guan's method; (**c**) Experimental data processed by the proposed method.

The experiment parameters are shown in Table 2. We first acquire the data according to the traditional sampling. Then, the range compression and range cell migration are applied to the echo data with the traditional approaches [20,39], and the corresponding result shown in Figure 10a. Figure 10b,c present the angular super-resolution results using the Guan's method and the proposed method, respectively. It can be seen that the proposed method has a better performance in terms of angular resolution. This is the fact that the TSVD method is able to suppress the noise amplification. We can also see that the angular super-resolution result obtained using the Guan's method can not resolve the reflectors, which locates in the same range cell but different angular cells. Therefore, we believe that the TSVD method for angular super-resolution in aircraft landing is useful in real applications.

## 5. Conclusions

A forward looking scanning radar imaging method using truncated singular value decomposition and its application for adverse weather aircraft landing are presented in this paper. After presenting and analyzing the signal model of forward looking scanning radar, we first convert the angular super-resolution imaging task into an equivalent deconvolution problem. In order to overcome the ill-posed nature of the deconvolution problem, we chose to compute a corresponding approximate solution by chopping off those SVD components that are dominated by the noise. The selection of the truncation parameter is based on GCV method. Compared with the Guan's method, the presented method improves the angular super-resolution performance in terms of precision. Simulation results indicate that the proposed method is effective in improving the angular resolution of scanning radar. The proposed method can also be applied to other inverse problem that are based on deconvolution. However, there are still some issues needed to be studied in angular super-resolution based on deconvlution method. Future work will study how to chose the truncation parameter adaptively and study the robustness of the deconvolution algorithm for angular super-resolution under low SNR levels.

## Figures and Tables

**Table 1:** Simulation parameters.

**Parameters**	**Value**	**Units**
Carrier frequency	10	GHz
Band width	75	MHz
Pulse duration	2	μs
Pulse repetition frequency	1000	Hz
Antenna scanning velocity	30	°/s
Antenna scanning area	−10 ∼+10	°
Main-lobe beam width	3	°

**Table 2:** Experimental Parameters.

**Parameters**	**Value**	**Units**
Carrier frequency	30.75	GHz
Band width	40	MHz
Pulse duration	2	μs
Pulse repetition frequency	4000	Hz
Antenna scanning velocity	60	°/s
Main-lobe beam width	4	°
Radar platform height	200	*m*
Antenna scanning area	−35∼+35	°
